# Supplementary material for: FastViromeExplorer: a pipeline for virus and phage identification and abundance profiling in metagenomics data
Source: PeerJ. 2018 Jan 12;6:e4227. doi: 10.7717/peerj.4227 (PMC5768174; doi:10.7717/peerj.4227)
Supplement: Supplemental Information 1 [file peerj-06-4227-s003.pdf]

Supplementary Figures
FastViromeExplorer: A Pipeline for Virus and Phage Identification and Abundance Profiling in Metagenomics Data

Saima Sultana Tithi[1], Frank O. Aylward[2], Roderick V. Jensen[2], Liqing Zhang[1*]
[1]Department of Computer Science, Virginia Tech, Blacksburg, VA 24061
[2]Department of Biological Sciences, Virginia Tech, Blacksburg, VA 24061
[*]Corresponding author: lqzhang@vt.edu

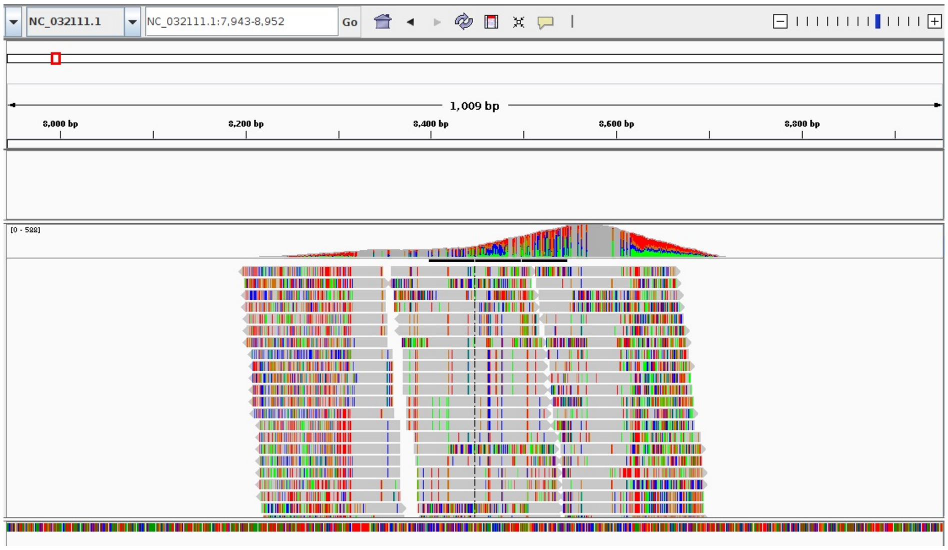

Supplemental Figure 1: Visualization of the reads mapped to the repeat region of BeAn 58058 virus

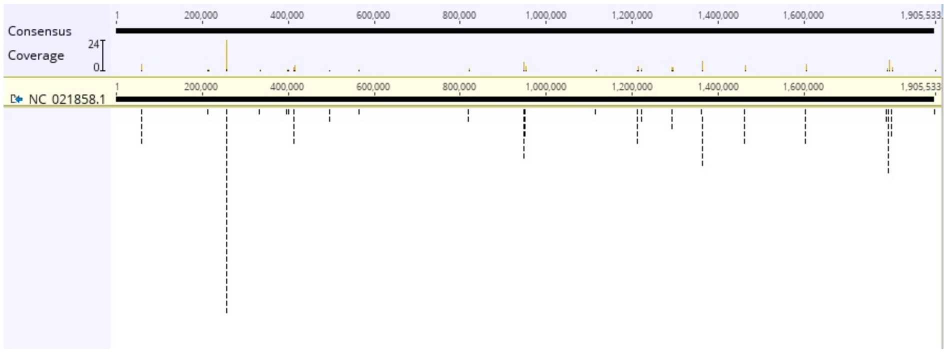

Supplemental Figure 2: Visualization of the reads mapped to the several repeat regions of Pandoravirus dulcis

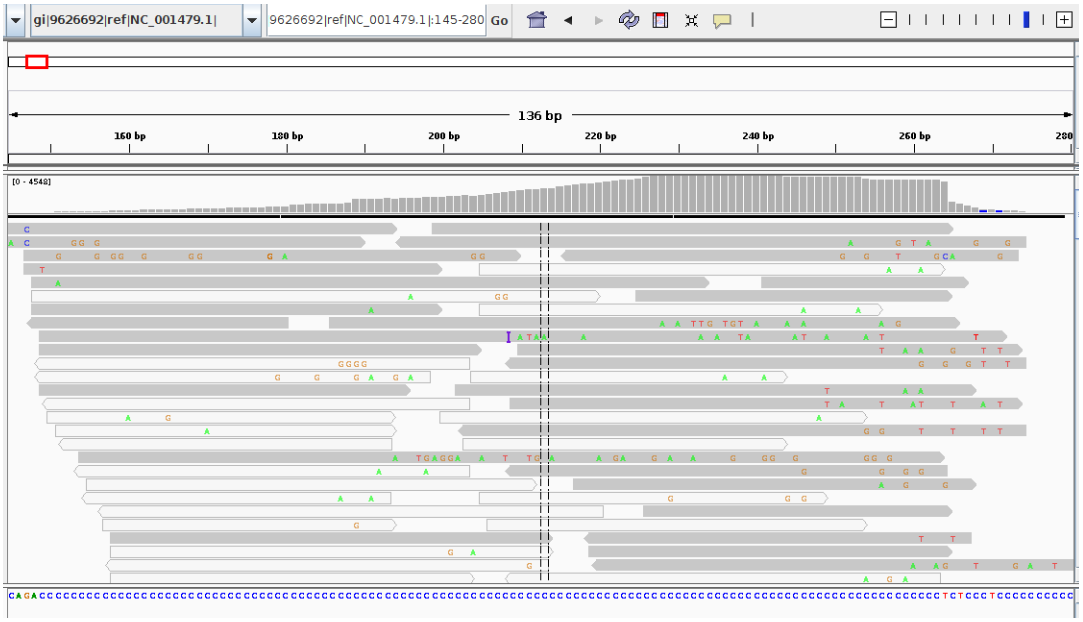

Supplemental Figure 3: Visualization of the reads mapped to the repeat region of Encephalomyocarditis virus from ViromeScan result
